# Clinical Outcomes of Spatially Fractionated GRID Radiotherapy in the Treatment of Bulky Tumors of the Head and Neck

**DOI:** 10.7759/cureus.4637

**Published:** 2019-05-10

**Authors:** J. Isabelle Choi, Janeen Daniels, Dane Cohen, Ying Li, Chul S Ha, Tony Y Eng

**Affiliations:** 1 Radiation Oncology, New York Proton Center, New York, USA; 2 Radiation Oncology, Eastern Maine Medical Center, Brewer, USA; 3 Radiation Oncology, University of Texas Health Science Center at San Antonio, San Antonio, USA; 4 Radiation Oncology, Emory University School of Medicine, Atlanta, USA

**Keywords:** sfgrt, spatially fractionated, bulky tumors, grid

## Abstract

Objectives

The clinical outcomes of patients treated with spatially fractionated GRID radiotherapy (SFGRT) for bulky tumors of the head and neck at a single institution were evaluated retrospectively. Endpoints of interest included tumor response, symptom improvement, treatment tolerance, and adverse events.

Methods

Institutional review board approval was obtained prior to study initiation. The institutional database was queried for patients with tumors of the head and neck treated with SFGRT between August 2007 and April 2015. Medical records of identified patients were reviewed for treatment details and clinical endpoints of interest. SFGRT was delivered in one fraction of 15 gray (Gy) or 20 Gy; 6 megavolt (MV) or 18 MV photon beams were passed through a multileaf collimator (MLC)-based or brass GRID template. All patients had a planned course of conventionally-fractionated external beam radiotherapy (EBRT) to begin on the day following SFGRT delivery.

Results

Twenty-one consecutive patients meeting study criteria were identified. The most common tumor histology was squamous cell carcinoma. Median patient age was 59 years (range 13 - 83 years); median maximum tumor dimension was 9.5 centimeters (cm) (range 5.0 - 25.0 cm). Fifteen patients (71.4%) completed their full course of EBRT. Twelve patients were treated with palliative intent for local tumor symptoms, of which 54.5% experienced some degree of symptom improvement. Of nine patients treated with curative intent, 44.4% achieved a clinical complete response (CR). Concurrent chemotherapy was administered in 12 patients, with all patients being treated having definitively received chemotherapy. Radiation Therapy Oncology Group (RTOG) grade three or higher skin toxicity occurred in five patients; no grade five events were reported.

Conclusions

Our institutional experience suggests that SFGRT is a feasible treatment option for the palliative or definitive management of large tumors of the head and neck. In combination with EBRT, SFGRT can provide timely symptom management and improve patient quality of life in the palliative setting. In the definitive setting, the addition of chemotherapy to SFGRT and EBRT can result in an excellent clinical response. Treatment toxicity was found to be within an acceptable range. When considering SFGRT for patients with these challenging presentations, careful patient selection is needed to identify those who will likely tolerate a full course of EBRT following SFGRT, as these patients are most likely to receive maximal benefit from SFGRT treatment. More data on the feasibility and efficacy of this radiation modality will be helpful for continued optimization of SFGRT delivery and patient selection.

## Introduction

The treatment of large solid tumors, whether in the setting of palliation or definitive cure, poses a significant clinical challenge. Surgical debulking or resection is often not a viable option given the associated morbidity of such a procedure, and other treatments for local tumor control are limited. Radiotherapy in various forms is an oft-used alternate treatment modality, but the probability of tumor control with conventionally fractionated external beam radiotherapy (EBRT) is limited in the setting of substantial tumor volume for most histologies [1-2]. Brachytherapy is a useful local treatment option given the limited associated normal tissue exposure to radiation and the potential for dose escalation, but this technique is frequently limited by tumor location, as well as clinical resources in the form of physician or medical physicist training and equipment not readily available in many centers.

An alternate method for the delivery of high dose radiotherapy is spatially fractionated GRID radiotherapy (SFGRT). With this technique, radiation is delivered through evenly spaced holes in a grid template overlying the tumor, resulting in a dose distribution similar to that of interstitial brachytherapy; however, in lieu of physical catheters, external pencil beamlets send the dose through the holes in the grid template to the tumor volume. Radiation is usually delivered in a single large fraction, which allows for dose escalation via an external beam “boost” to the tumor. Adjacent normal structures are also spared using this method as minimal or no clinical or planning margin is added to the tumor volume, in an approach akin to stereotactic radiosurgery [3].

The advantage of this method of external dose delivery was first recognized when orthovoltage radiation was a dominant method of external beam radiation delivery. Dose escalation with orthogonal x-rays is limited by skin toxicity due to a high surface dose delivered over large fields. SFGRT was developed to supplement the tumor dose, made possible due to its skin-sparing effects [4]. With the development of modern megavoltage radiation therapy with which superficial dose is less limiting, the need for SFGRT has diminished. However, SFGRT does still hold relevance in modern-day radiation therapy for select cases, such as with large tumors for which conventionally fractionated EBRT alone would be of limited benefit and interstitial brachytherapy is not a feasible or accessible option.

At our institution, SFGRT is used in select patients who present with large tumors, most often in the head and neck region, to supplement the dose delivered by EBRT and in an attempt to provide more timely symptom relief. In this study, we identify these cases and analyze clinical endpoints of treatment tolerance, tumor response, symptom improvement, and treatment-related toxicity.

## Materials and methods

Patient selection

Institutional Review Board approval was obtained prior to study initiation. A search was performed in the institutional electronic medical record system to identify all patients treated with SFGRT from August 2007 to April 2015. The radiotherapy plans and treatment records of these patients were reviewed. Radiotherapy treatment details were collected to include SFGRT treatment and conventionally fractionated EBRT delivered, if any. Endpoints of interest included tumor response, symptom improvement, treatment tolerance, and adverse events. All patients signed informed consent prior to the delivery of radiation therapy. 

SFGRT technique

**Figure 1:**
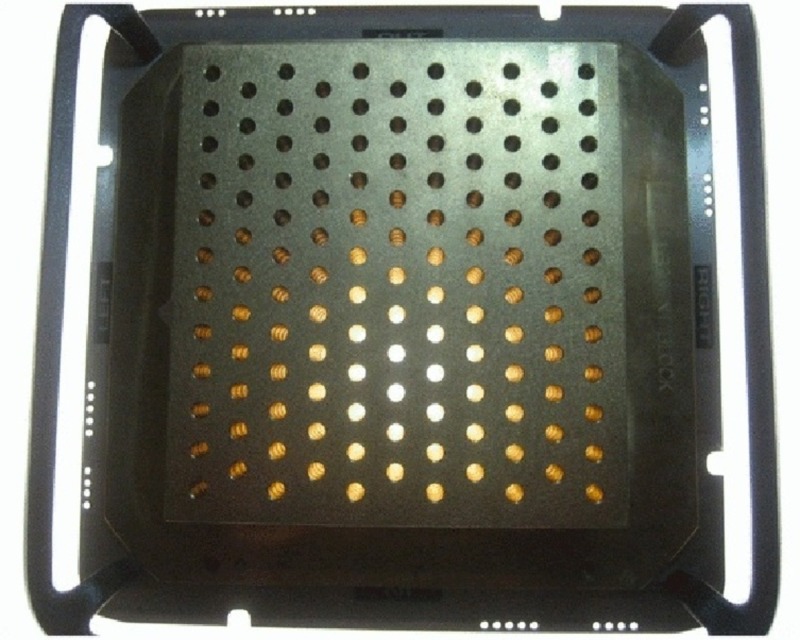
Example of a brass MLC-based GRID used in SFGRT treatment. Photon beamlets pass through evenly spaced holes in the GRID. Each hole is 1 cm in diameter, with a distance from center to center of 2 cm. The brass block is secured on a metal tray and placed in the location where an IMRT solid compensator would be placed. MLC = multileaf collimator; SFGRT = spatially fractionated GRID radiotherapy; cm = centimeter; IMRT = intensity-modulated radiotherapy

Patients who received SFGRT prior to 2008 were treated using a multileaf collimator (MLC)-based GRID. The GRID fields were designed by creating custom MLC patterns with different open to blocked area ratios and with variable separation between the openings. Patients treated after 2009 were treated using a commercially produced universal brass GRID block manufactured by .decimal (.decimal Inc., Sanford, FL). The brass block is secured on a metal tray placed in the same location as a regular intensity-modulated radiotherapy (IMRT) solid compensator. The holes of the brass block are 2 centimeters (cm) from center to center and arranged in a hexagonal array. Each hole is divergent and projects a 1 cm circular field at the isocenter (Figure 1). The GRID design was imported into the Pinnacle treatment planning system. Dosimetric characteristics using an MLC-based and universal brass GRID block have been previously described [3,5].

**Figure 2:**
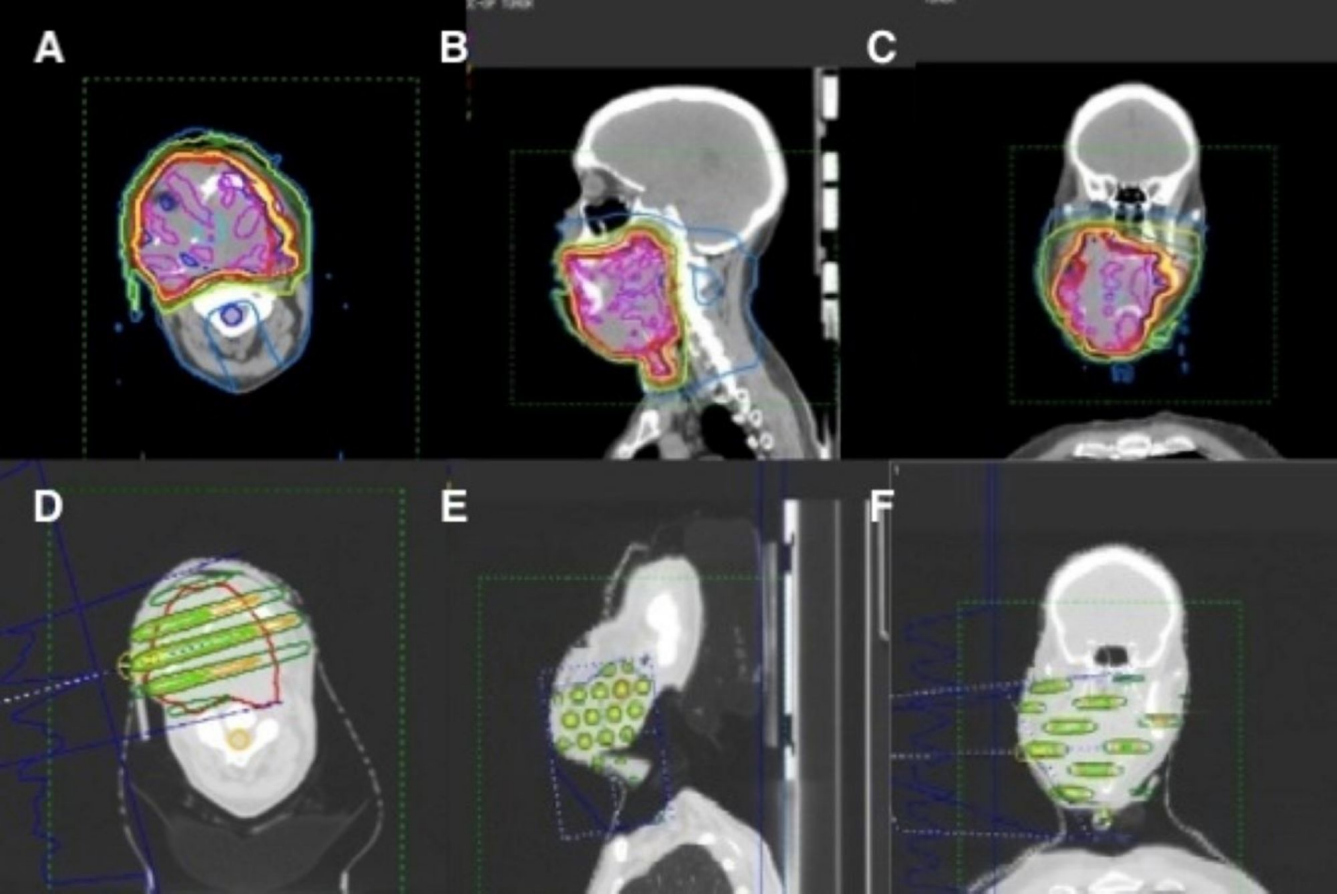
Representative images of a radiotherapy plan for one patient with bulky recurrent disease of the head and neck who was treated at our center with SFGRT (15 Gy in 1 fraction) followed by IMRT (4080 cGy delivered in 34 fractions given twice daily). A-C. Isodose distribution of IMRT plan (Magenta = 4243 cGy; Blue = 4080 cGy; Yellow = 3876 cGy; Red = 3672 cGy; Green = 2856 cGy; White = 2040 cGy; Teal = 400 cGy). D-F. Isodose distribution of SFGRT plan (Blue = 1800 cGy; Yellow = 1500 cGy; Light Green = 1000 cGy; Orange = 800 cGy; Dark Green = 400 cGy). A. & D. Axial view; B. & E. Sagittal view; C. & F. Coronal view. SFGRT = spatially-fractionated GRID radiotherapy; Gy = gray; IMRT = intensity-modulated radiotherapy; cGy = centigray

Patients were treated with SFGRT in a single fraction of 15 gray (Gy) prescribed to the depth of maximum dose (Dmax) using a source to surface distance (SSD) of 100 cm, except from 2010 to 2011, when patients were treated in a single fraction of 20 Gy, also prescribed as above. Six MV or 18 MV photons were used to treat most patients, depending on depth and optimal dosimetric coverage. The beam’s central axis was aligned with the center of the tumor volume and was positioned under the centermost hole of the compensator block. Target delineation involved the contouring of gross tumor volume (GTV), as well as organs-at-risk, including the brainstem, spinal cord, orbits, lenses, optic nerves, optic chiasm, cochlea, and esophagus, as pertinent to each case. One tangential field encompassing the GTV with autoblocks around the volume was designed. An appropriate beam angle was chosen for optimal tumor volume coverage, with concomitant maximal avoidance of critical normal structures (i.e., mandible, spinal cord, brainstem, brain, and brachial plexus) and traversing of the smallest skin to GTV separation possible. No additional expansion on the GTV was made. An example GRID treatment plan is demonstrated in Figure 2.

CT simulation was performed with the use of a custom thermoplastic Orfit head and neck mask (Orfit Industries, Belgium) with a MOLDCARE® cushion (Qfix, Avondale, Pennsylvania) if tolerated by the patient.

EBRT treatment

SFGRT was followed on the next day with the initiation of a course of fractionated external beam radiation therapy, the majority of which were delivered via IMRT (Figure 2). Dose and fractionation varied widely, depending on several factors, to include patient performance status, goal of treatment (palliative versus definitive), and tumor size.

Systemic therapy

Chemotherapy was delivered at the discretion of each patient’s primary medical oncologist. If given, this was initiated concurrently with the first fraction of EBRT after SFGRT delivery.

Patient follow-up

Patients were monitored weekly during radiation therapy and acute toxicities were reported. Patients were followed until death, enrollment into hospice, or loss of follow-up. Treatment response, including change in tumor size, symptom improvement, and local control were reviewed.

## Results

Patient characteristics

We identified 21 consecutive patients treated with SFGRT at our institution between August 2007 and April 2015. Patient characteristics are listed in Table 1. All patients presented with large tumors (>5 cm) in the head and neck region. The most common tumor histology was squamous cell carcinoma (SCC) arising from a primary head and neck subsite or in a neck nodal metastasis (16/21 patients). Other histologies included anaplastic carcinoma of the left face, poorly differentiated invasive carcinoma in a metastatic neck node (likely skin primary), osteosarcoma of the mandible, pleomorphic sarcoma in a previously irradiated oropharynx field, and malignant peripheral nerve sheath tumor (MPNST) of the right neck. Patient ages ranged from 13 to 83 years, with a median age of 59 years. Maximum tumor dimension ranged from 5.0 to 25.0 cm, with a median diameter of 9.5 cm.

**Table 1:** Patient characteristics SCC = squamous cell carcinoma; cm = centimeter; n = number

Patient Characteristics	Definitive	Palliative	All Patients
Median Patient Age (years) (Range)	59 (52-64)	59 (13-83)	59 (13-83)
Median Performance Status (Range)	80 (60-90)	75 (50-100)	80 (50-100)
Median Tumor Size (cm) (Range)	9 (5-14)	10 (6.8-25)	10 (5-25)
SCC Histology (n)	9	7	16
Non-SCC Histology (n)	0	5	5

Twelve of 21 patients (57.1%) were treated with palliative intent to improve local tumor symptoms, including bleeding (n=2), restricted motion from tumor bulk (n=11), and pain (n=3). Nine of 21 patients (42.9%) were treated with curative intent. Median performance status, age, and maximum tumor dimension were similar between the two groups, although ranges were wider in the palliative group. Non-SCC histology was only seen in the palliative group.

Radiotherapy treatment

SFGRT was delivered prior to any planned conventionally fractionated EBRT course. Five of 21 patients received SFGRT to 20 Gy in one fraction while the remaining 16 patients received 15 Gy in one fraction. EBRT typically began one to three days after SFGRT delivery. EBRT dose-fractionation prescriptions varied widely depending on the goal of treatment, patient performance status, and prior history of radiotherapy to the region. For those patients being treated with curative intent, all were prescribed EBRT via IMRT to 69.96 to 72.08 Gy in 2.12 Gy daily fractions. Palliative doses ranged from 25 Gy in 10 fractions to 78 Gy in 39 fractions. Additional treatment details can be found in Table 2.

**Table 2:** Treatment characteristics SFGRT = spatially-fractionated GRID radiotherapy; Gy = gray; n = number; MV = megavolt; EBRT = external beam radiotherapy

Treatment	Definitive	Palliative	All Patients
SFGRT			
Dose			
15 Gy (n)	9	7	16
20 Gy (n)	0	5	5
Beam Energy			
6 MV (n)	9	7	16
18 MV (n)	0	5	5
EBRT			
Median Dose Prescribed	69.96 Gy	50.6 Gy	69.96 Gy
(Range)	(69.96 – 72.08 Gy)	(25 – 78 Gy)	
Median Dose Delivered	69.96 Gy	46.0 Gy	69.96 Gy
(Range)	(2.12 – 72.08 Gy)	(2.5 – 78 Gy)	
Mean Dose Prescribed	70.2 Gy	54.5 Gy	61.6 Gy
Mean Dose Delivered	62.7 Gy	41.5 Gy	51.0 Gy
Chemotherapy (received, n)	9	3	12

Systemic therapy

Twelve of 21 (57%) patients received concurrent chemoradiation. Cisplatin was given in nine of 12 patients, either as a single agent (four patients) or in a doublet with paclitaxel (four patients) or etoposide (one patient) (Tables 3-4) Single agent cetuximab was used in two patients, and paclitaxel in one patient. All nine patients treated with curative intent received concurrent chemotherapy.

**Table 3:** Clinical response in patients treated with palliative intent Pt = patient; No = number; EBRT = external beam radiotherapy; SCC = squamous cell carcinoma; PR = partial response; n/a = not applicable; MPNST = malignant peripheral nerve sheath tumor

Pt No.	Histology	Received >75% EBRT Rx	Concurrent Chemotherapy	Symptom Improvement	Tumor Response	Reirradiation
1	Anaplastic carcinoma	No	Cisplatin/etoposide	No (size)	None	
2	Poorly differentiated carcinoma	No		Yes (pain, bleeding)	N/A	
3	SCC	No		N/A	N/A	
4	Pleomorphic sarcoma	Yes		Yes (size)	Less than PR	Yes
5	SCC	Yes		No (size)	None	
6	SCC	Yes	Cetuximab	Yes (size)	Less than PR	
7	Osteosarcoma	Yes		No (size)	None	
8	SCC	No		No (size)	None	
9	SCC	Yes		Yes (size)	Less than PR	
10	SCC	Yes		Yes (pain, bleeding)	PR	Yes
11	SCC	No	Paclitaxel	No (size)	None	
12	MPNST	Yes		Yes (size)	Less than PR	

**Table 4:** Clinical response in patients being treated with definitive intent Pt = patient; No = number; EBRT = external beam radiotherapy; SCC = squamous cell carcinoma; PR = partial response; CR = complete response

Pt No.	HIstology	Received >75% EBRT Rx	Concurrent Chemotherapy	Tumor Response	Additional Therapy
13	SCC	No	Cetuximab	None	
14	SCC	Yes	Cisplatin/paclitaxel	CR	
15	SCC	Yes	Cisplatin/paclitaxel	CR	
16	SCC	Yes	Cisplatin/paclitaxel	CR	
17	SCC	Yes	Cisplatin	Less than PR	Neck dissection
18	SCC	Yes	Cisplatin	None	
19	SCC	Yes	Cisplatin	PR	
20	SCC	Yes	Cisplatin	CR	
21	SCC	Yes	Cisplatin/paclitaxel	Less than PR	Neck dissection

Treatment completion

Overall, 15 of 21 patients (71.4%) completed their full planned course of EBRT. Of the six patients who did not complete treatment, with the exception of one patient, all were being treated for palliation. Five out of 21 patients (23.8%) completed <50% of the planned EBRT course due to patient preference (two patients), progression (one patient), or clinical deterioration (two patients). One patient did not start their EBRT course and was admitted to hospice due to a rapidly deteriorating clinical state. 

Response rate

Clinical response was evaluated by assessing tumor shrinkage and improvement in local symptoms caused by tumor burden (11 evaluable patients). A partial tumor response (PR) was defined as a greater than 50% reduction in tumor maximum diameter. Of patients being treated for palliation, only one patient of 10 with these data available achieved a PR (Table 3). Some degree of tumor response was seen in four additional patients but not enough to be considered a PR.

Improvement of discomfort due to tumor bulk was achieved in four of nine patients being treated for this issue. Both patients being treated for palliation of pain and bleeding from local tumor invasion experienced relief from treatment. Of patients who received less than 75% of their radiation dose, only 25% experienced symptom improvement. For patients with non-SCC histologies, 60% achieved some degree of palliation after SFGRT. Overall, six of 11 patients (54.5%) received some degree of palliation from SFGRT. In patients with SCC histology and who received >75% of their EBRT dose, a 75% palliation rate was achieved.

In patients being treated definitively, four of nine patients (44.4%) achieved a clinical complete response to treatment (Table 4). One patient achieved a PR, and two patients had less than PR after treatment. Of the two patients who had no response to therapy, one patient only completed one out of planned 33 fractions of EBRT after SFGRT. The other patient without a clinical response and one patient with less than PR underwent surgical neck dissection after completion of therapy. All patients had a diagnosis of SCC of the head/neck region, and all were treated with concurrent chemotherapy.

Survival

Five patients were lost to follow-up and, therefore, do not have survival data available. The date of the last data analysis was July 31, 2015, with follow-up ranging from one week to 16 months. If a patient transitioned to hospice care during EBRT (three patients), it was assumed the patient passed away within two months. At the time of the last follow-up, nine of 16 patients with available data succumbed to their disease. Of these patients, two were being treated with definitive intent. Of the seven patients treated with palliative intent with follow-up survival data available, all had died at the time of the last analysis, with a median time to death of two months (range one week - nine months). Follow-up data were available for all patients treated with definitive intent. Seven of the nine patients treated definitively were still alive at the time of the last analysis. Death occurred four months and 17 months after completion of treatment, respectively, for the two patients who died. For the seven patients who are still alive, the median follow-up is seven months (range 4 - 16 months).

Toxicity

Skin and mucous membrane treatment toxicity were graded using the Radiation Therapy Oncology Group (RTOG) Acute Morbidity Scoring Criteria. Grade three or higher skin toxicity was noted in five patients, including four cases of grade four toxicity (Table 5). Three patients developed bleeding, with two patients requiring hospitalization (n=2) or embolization (n=1) for this issue while the fourth patient developed skin ulceration from tumor regression. No grade three or higher mucous membrane toxicities were observed, and no grade five toxicity was recorded. Late toxicities are not reported in this initial analysis given the short interval follow-up.

**Table 5:** Observed toxicities graded per RTOG acute morbidity scoring criteria RTOG = Radiation Therapy Oncology Group; n = number

RTOG Acute Toxicity	Skin (n)	Mucous Membrane (n)
Grade 0	3	7
Grade 1	4	0
Grade 2	6	10
Grade 3	1	0
Grade 4	4	0

## Discussion

Bulky tumors of the head and neck pose a unique treatment challenge as EBRT, either in conventionally fractionated or hypofractionated regimens, results in suboptimal clinical response rates. Dose escalation via high-dose external beamlets results in a biologic effect akin to that of interstitial brachytherapy, which, when used in combination with conventionally fractionated EBRT, can allow for improved local tumor control and better symptom palliation while simultaneously limiting toxicity to the skin and normal adjacent structures. This is the premise of SFGRT, in which a GRID block is placed over the tumor and a high dose of radiation is delivered through small, evenly spaced holes in the GRID template.

Various models of the GRID are available, including the Cerrobend GRID block, brass GRID block, and the MLC-based GRID. The non-MLC GRID block is designed to fit in the wedge tray or block tray of the treatment linear accelerator. In general, one or two tangential fields are used for treatment via a 2D technique. The deep border encompasses the tumor GTV without additional margin, in an approach analogous to that of brachytherapy or SBRT. The dose is delivered most commonly in one fraction of 10-20 Gy [6-9].

The rationale behind the radiobiological advantage of SFGRT remains incompletely understood. Several theories have been proposed to help elucidate the mechanism by which SFGRT can provide added therapeutic benefit to conventional treatment methods. In a study of 114 tumors in patients treated for cervical cancer, Zhang et al. used the linear-quadratic (L-Q) model to calculate the equivalent open-field dose and therapeutic ratio (TR) of SFGRT with a hexagonal GRID with either a conventionally-fractionated (2 Gy per fraction) or hypofractionated (15 Gy in one fraction) regimen [10]. He found that SFGRT resulted in an improved TR for both conventionally fractionated and hypofractionated regimens versus an open field technique, with a greater benefit seen for hypofractionation. It was hypothesized that due to improved normal tissue sparing with SFGRT, along with a therapeutic advantage in treating acutely responding tumor tissue with a higher dose per fraction, the addition of SFGRT to a course of conventionally fractionated radiotherapy resulted in overall improved outcomes. This finding was also validated in a study of melanoma tissues, in which the Monte Carlo method was used to model treatment with SFGRT and evaluate survival statistics [11]. Zhang et al. found that treatment with SFGRT resulted in favorable outcomes for radiosensitive tumor tissue, without a similar benefit seen in late-responding tissue.

The bystander effect has also been implicated as a possible mechanism by which SFGRT promotes increased tumor response versus fractionation with smaller doses per fraction. The possible methods of an increase in regional damage due to the bystander effect include cell-to-cell communication, free radical damage, change in gene expression, and cytokine release. In a 2002 study performed by Sathishkumar et al., the serum levels of cytokines were measured in 31 patients after the treatment of 34 sites [12]. Cytokines of interest were TNFα and TGF-β1, both known to be released in response to system stress such as that caused by high dose radiotherapy treatment. They found that those patients who experienced an increase in serum levels of cytokine TNF-alpha were also more likely to achieve a complete clinical response to therapy. The increased release of cytokines from a single large fraction of radiation as given with SFGRT may result in increased regional tumor cell kill and thus improved tumor response and control as compared with more fractionated treatment.

The use of SFGRT is still limited to relatively few radiation therapy centers in the U.S. Possible explanations for this include limited published efficacy and safety data, technical challenges, lack of equipment for implementation, and limitations in facility personnel experience, and, therefore, comfort, in delivering this type of treatment. For a majority of the patients treated in this study, a commercially available brass GRID template (.decimal, Inc., Sanford, FL) was used. The effective dosimetry of the brass GRID template was validated in a study by Buckey et al. [3]. With the increased availability of commercially available GRID blocks and demonstrated clinical ease of use, more centers may be encouraged to implement their use.

In recent years, several institutional experiences have been published establishing the technical feasibility and clinical utility of SFGRT. In a retrospective review by Neuner et al. [6], patients treated at the University of Maryland with Cerrobend or MLC-based SFGRT experienced a 75% response rate in palliation of pain and 70% improvement in mass effects, with acceptable toxicity rates. They noted a dose-dependent relationship, with the addition of SFGRT as an adjunct to external beam radiation therapy. Similarly, Peñagaricano et al., at the University of Arkansas, published their institutional review of 14 patients with bulky (6-8 cm) tumors of the head and neck treated with MLC-based SFGRT and external beam radiotherapy with simultaneous integrated boost (SIB)-IMRT, both concurrent with chemotherapy [8]. They found an 80% pathologic complete response rate in patients who underwent a planned neck dissection or primary tumor biopsy after radiotherapy and a similar 79% combined pathologic and clinical complete response rate for all patients. Toxicity was comparable to that previously established for treatment without the addition of SFGRT [8]. These findings are reflected in this study, which demonstrates a 70% improvement in palliation of symptoms for patients who received >75% of their EBRT course. An 87.5% overall response rate was seen in patients being treated with definitive intent who received >75% of their EBRT prescription dose, with 50% achieving a complete clinical response.

Our institutional review suggests that SFGRT can be an effective radiation therapy modality for large, symptomatic tumors that can provide timely improvement in patient quality of life and improve response rates in the definitive setting. At initial analysis, the incidence of severe skin toxicity with the addition of SFGRT does appear to be substantial. However, when taking into account the inherent invasiveness of these head and neck tumors, often with compromise of the overlying skin and surrounding vasculature at presentation, ulceration and bleeding seen concomitantly with the regression of these tumors in response to therapy are likely the natural course of tumor response, instead of a disparate effect of treatment.

Patients who were unable to complete their full course of EBRT had predictably worse outcomes, likely due to a combination of inadequate treatment, radiation-resistant tumor types, and baseline poor performance status, which independently predicts for poor outcomes. Therefore, the most pragmatic implementation of SFGRT in the clinic may be as an adjunct treatment for those patients who have an adequate pre-treatment performance status and a high likelihood of treatment completion. For those patients with poor performance status at presentation or for tumors exhibiting signs of treatment resistance, the use of SFGRT for fast palliation, regardless of the extent of fractionated EBRT that can be delivered after SFGRT, could still play a useful role in symptom improvement and maximization of quality of life.

The use of this modality is relatively limited, likely in part due to the small body of clinical outcomes data reported on SFGRT to date. We show in our study that SFGRT can be a valuable tool in the radiation oncology clinic when presented with the challenging situation of a patient with an inoperable tumor of sizeable bulk that would likely experience limited response and control with conventionally fractionated radiotherapy alone. With more commercially available SFGRT delivery systems and continued validation of these technologies, this resource can be implemented in more centers. In addition, SFGRT has been used in other tumor sites at a number of centers; in the future, continued application of SFGRT to sites that would be otherwise considered for interstitial brachytherapy, such as the extremities, will be of interest. With more experience, additional improvements in treatment planning and delivery are expected, such as in dose optimization, timing of delivery, and beam setup.

## Conclusions

With careful patient selection, SFGRT is a useful clinical tool in both the definitive and palliative management of bulky, unresectable tumors of the head and neck, with an acceptable toxicity profile. Continued investigations will further elucidate the role of SFGRT in select clinical situations. In addition, the potential for combining immunotherapy with the high radiation doses delivered with SFGRT is of particular interest and would benefit from a further prospective study.
